# Upstream – News in Genomics

**DOI:** 10.1002/cfg.125

**Published:** 2001-12

## Abstract

Since our last issue, several important genomes have been completely or ‘almost completely’ sequenced. The debate over the number of human genes has flared up once more, with one computational and one experimental study into the annotation of the human genome. The mouse genome project has a clone fingerprint map to aid their sequencing effort. The SAGE technique has been applied to *Drosophila* and the US National Science Foundation announced increased spending on plant genome research.


**Comparative and Functional Genomics**
*Comp Funct Genom* 2001; **2**: 355–358.
**DOI:** 10.1002/cfg.125

**Feature**

### Upstream – news in genomics

### Abstract

**Since our last issue, several important genomes have been completely or 'almost completely' sequenced. The debate over the number of human genes has flared up once more, with one computational and one experimental study into the annotation of the human genome. The mouse genome project has a clone fingerprint map to aid their sequencing effort. The SAGE technique has been applied to *Drosophila* and the US National Science Foundation announced increased spending on plant genome research. Copyright © 2001 John Wiley & Sons, Ltd.**

Published online:
14 November 2001

### Genome sequencing

On August 31, a team from the National Institute of Technology and Evaluation, in Tokyo published the complete **genome sequence of an aerobic thermoacidophilic crenarchaeon, Sulfolobus tokodaii strain7** (Kawarabayasi *et al.*, 2001). *S. tokodaii* grows optimally at 80°C, at low pH, and under aerobic conditions. The genome is 2.69 Mb with a G+C content of 32.8%, and contains 2826 potential protein-coding regions, or open reading frames (ORFs). 911 ORFs are related to genes that have been assigned a function, 921 are related to conserved ORFs with unknown function, 145 have some motifs, and the remaining 849 showed no significant similarity to any sequences. Comparative studies suggested that the integration of a plasmid, rearrangement of genomic structure, and duplication of certain genomic regions might be the cause of the larger genome size of *S. tokodaii* strain7. The team found eukaryote-like genes, which have not been identified in other archaea, and saw that the tRNA genes lack the CCA sequence. They conclude that this strain could be more closely related to eukaryotes than the other archaea sequenced so far.

The **complete genome of Yersinia pestis**, the causative bacteria of the Plague (also known as the Black Death), was published on October 4 by a team from the Wellcome Trust Sanger Institute led by Julian Parkhill (Parkhill *et al.*, 2001). The genome of strain CO92, was shown to consist of a 4.65 Mb chromosome and three plasmids (of 96.2 kb, 70.3 kb and 9.6 kb). *Y. pestis* appears to have evolved from a fairly benign intestinal bacterium, *Y. pseudotuberculosis*. Its hosts are the fleas that live on black rats, and if these fleas bite a human host, it is able then to live in the blood, causing swelling, coughing and haemorrhaging that can be fatal. In the transition to this lifestyle, it appears to have acquired genes from viruses and other bacteria, including those for the toxins that enable it to infect fleas, and there are around 150 pseudogenes (several of which are the remnants of genes that were no longer needed once it had abandoned the enteropathogenic lifestyle).

On October 24, an international consortium announced their **completion of a draft genome sequence of Fugu rubripes**, the puffer fish. This has been a collaborative effort between the MRC Human Genome Mapping Project Resource Centre (HGMP-RC), the DOE Joint Genome Institute, the Institute for Molecular and Cell Biology in Singapore and the Institute for Systems Biology. It has taken just under a year for the team to complete a draft of this ~365 Mb vertebrate genome. The draft consists of ~20 000 contigs, covering 310 Mb of unique DNA sequence. They estimate that this covers ~90% of the non-repetitive fraction of the genome. Whilst the *F. rubripes* genome is expected to have a broadly similar gene content to human, it has far less repeat sequences and significantly shorter intergene and intron sequences (typically around 7-fold shorter). Comparisons of *F. rubripes* and human sequence can aid gene detection by identifying conserved exons and, further, can identify promoter regions, which are much harder to detect by automated approaches.

In the October 25 issue of *Nature*, researchers from the Sidney Kimmel Cancer Center, California, published the **genome of Salmonella enterica subspecies I, serovar Typhimurium** (*S. typhimurium*, McClelland *et al.*, 2001), a leading cause of human gastroenteritis. Strain LT2 has a 4.86 Mb genome and a 94 kb virulence plasmid. Using the previously

Copyright © 2001 John Wiley & Sons, Ltd.

completed genomes of three related bacteria, sample sequencing of *S. enterica serovar Paratyphi A* (*S. paratyphi A*) and *Klebsiella pneumoniae*, and hybridization analysis of three unsequenced genomes to a microarray of *S. typhimurium* LT2 genes, the distributions of close homologues to *S. typhimurium* LT2 genes in these related bacteria were determined. Lateral transfer of genes appears common, 11% of the *S. typhimurium* LT2 genes are missing from *S. enterica serovar Typhi* (*S. typhi*), and 29% are absent in *Escherichia coli* K12. The 352 LT2 homologues shown to be confined to subspecies I of *S. enterica* (a grouping containing most mammalian and bird pathogens) will be useful for studies of epidemiology, host specificity and pathogenesis. Most of these are novel, interestingly, 50 of them may be exported to the periplasm or outer membrane, making them good targets for therapeutics or vaccines.

The **genome of Salmonella enterica serovar Typhi** (*S. typhi)*, the related typhoid fever bacterium, was published in the same issue of *Nature* (Parkhill *et al.*, 2001). The ~4.8 Mb genome of the CT18 strain, which is resistant to all common antibiotics, and is close to becoming untreatable, was sequenced at the Wellcome Trust Sanger Institute, in collaboration with teams in Denmark and Vietnam. The majority of *S. enterica* serovars invade the mucosal surface of the intestine but are contained (in healthy individuals) by human immune defence mechanisms. *S. typhi* has evolved the ability to spread to the other tissues, including the liver, spleen and bone marrow. Compared with the *Escherichia coli* genome, *S. typhi* has hundreds of insertions and deletions, from single genes to large islands. There are 4599 genes, including 204 pseudogenes, some of which match genes that contribute to virulence in *Salmonella typhimurium*. This may partly explain the human-restricted host range for *S. typhi*. This strain has an ~218 kb multiple-drug-resistance plasmid (pHCM1), and a 106 kb cryptic plasmid (pHCM2), which appears to be a relative of a virulence plasmid in *Y. pestis*.

### Genome annotation

The glowing embers of the human gene count debate were fanned back into flame in July by the publication of **a new analysis of the human genome** (Wright *et al.*, 2001). A group from Ohio State University (OSU) performed their own annotation of the human genome, using a range of databases to identify potential exons. They claim that both of the existing annotations, that of Celera, and that of the Human Genome Mapping Project, have underestimated the number of human genes, because they used a more limited set of resources to aid gene identification. The OSU team combined the major public cDNA, EST and protein resources and resolved any redundancies, creating an exon index. They then located the exons uniquely on the genome, using BLAST (taking only the best matches to genomic sequence). They estimate that the genome contains 65 000–75 000 transcriptional units, with exon sequences comprising 4% of the total genome.

This was followed in September by **a new study with implications for the number of human genes** by researchers at McGill University, Montreal (Das *et al.*, 2001). The initial analysis of human chromosome 22 (Dunham *et al.*, 1999) identified 545 genes and 134 pseudogenes with similarity, or identity, to known proteins and/or ESTs. Scaling up from this analysis produced an estimate of ~36 000 expressed genes (and 9000 pseudogenes) in the human genome. However, hundreds of additional genes (beyond those annotated in the chromosome 22 sequence) were predicted by the gene prediction program Genscan. The McGill University group has used a sensitive RT-PCR assay, on samples from 17 tissues and one cell line, to determine how many of these 'predicted novel genes' (PNGs) represent expressed human genes. Their results indicate that there are at least 5000–9000 additional human genes (which lack similarity to known genes or proteins), which would increase low-end gene estimates to ~41 000–45 000.

On September 20, Ensembl released **Ensembl Mouse**, which displays a mouse physical map, comprised of 554 BAC contigs, constructed using clone fingerprinting data. Draft and finished sequence data are included in this resource and the predictions made by the Ensembl automated annotation tools are also provided. The map covers over 95% of the genome, with around 300 Mb of sequence (~10% of the genome). The site offers browsing by chromosome, BLAST searching against the contigs, or the peptide or cDNA predictions, or SSAHA (Sequence Search and Alignment by Hashing Algorithm) searches of the Arachne Whole Genome Shotgun assembly from the Whitehead Center for Genome Research.

In the October 9 issue of *PNAS*, a Brazilian group reported on their contribution to the growing

Copyright © 2001 John Wiley & Sons, Ltd.

catalogue of our transcriptome (Camargo *et al.*, 2001). They sequenced ~**700 000 ORESTES (Open Reading frame Expressed Sequence Tags**), ~560 000 of which were selected for analysis. ORESTES are short RT-PCR generated sections from the central region of mRNAs. These were generated from 24 normal and malignant samples, as part of the Fundação de Amparo à Pesquisa do Estado de São Paolo and Ludwig Institute for Cancer Research Human Cancer Genome Project. This data complements the vast arrays of 3′ and 5′ end sequences already available, providing scaffolds of partial sequences that can be used to obtain full-length data using RT-PCR and RACE reactions, and in some cases providing overlapping data that can be assembled into full-length sequences. ORESTES are also normalised for poorly expressed mRNAs, hence the approach shows much less bias towards moderately and highly expressed messages, compared to 5′ and 3′ end sequence collections.

### Functional genomics

In the October issue of *Developmental Cell*, researchers at the European Molecular Biology Laboratory in Heidelberg and the Center for Cancer Biology, at the University of Rochester Medical Center reported a **Serial Analysis of Gene Expression** (SAGE) study into the response of the **Drosophila melanogaster** embryo to Jun N-terminal kinase (JNK) signalling (Jasper *et al.* 2001). JNK regulates morphogenetic tissue closure movements that involve cell shape changes and reorganization of the actin cytoskeleton. Hundreds of genes were shown to respond to this signal, including genes encoding cell adhesion molecules and cytoskeletal regulators.

On November 1, the US **National Science Foundation (NSF) awarded 24 grants**, totalling $71 million towards **plant genome research** for 2002. This is a big increase on last year's figure, which was ~$48 million. The NSF's National Plant Genome Research Program was started in 1998 and was designed to build an understanding of the structure and function of plant genes important to agriculture, environmental management, energy and health. Examples of the projects chosen for support are the functional genomics of barley, mapping the tomato genome, studies of grass-genome diversity and whole-genome analysis of pathogen-host recognition in rice. There are also projects focussing on innovative methods for gene discovery and characterization, including the development of homologous gene replacement, massively parallel signature sequencing and mutations induced by transposons. A common theme amongst the successful applicants was multi-institutional academic collaborations concentrating on commercial crops such as rice, corn, and sorghum.

### Related websites

Ensembl Mouse
**http://mouse.ensembl.org/**

Initial chromosome 22 Annotation
**http://www.sanger.ac.uk/HGP/Chr22**

Institute for Systems Biology
**http://www.systemsbiology.org/**

Institute of Molecular and Cell Biology
**http://www.imcb.nus.edu.sg/**

Joint Genome Institute
**http://www.jgi.doe.gov**
JGI *Fugu* page – **http://www.jgi.doe.gov/fugu/index.html**

MRC HGMP
**http://www.hgmp.mrc.ac.uk**
HGMP *Fugu* page – **http://fugu.hgmp.mrc.ac.uk/**

NSF Plant Genome Research Program
**http://www.nsf.gov/bio/dbi/dbi_pgr.htm**
Complete awards listing – **http://www.nsf.gov/bio/pubs/awards/genome01.htm**

*S. tokodaii* genome data
**http://www.bio.nite.go.jp/E-home/genome_list-e.html/**

Wellcome Trust Sanger Institute
*Y. pestis* – **http://www.sanger.ac.uk/Projects/Y_pestis/**
*S. typhi* – **http://www.sanger.ac.uk/Projects/S_typhi/**

### References

Camargo AA, Samaia HPB, Dias-Neto E, *et al.* 2001. The contribution of 700 000 ORF sequence tags to the definition of the human transcriptome. *Proc Natl Acad Sci U S A* **98**: 12103–12108.

Das M, Burge CB, Park E, Colinas J, Pelletier J. 2001.

Copyright © 2001 John Wiley & Sons, Ltd. 

Assessment of the total number of human transcription units. *Genomics* **77**: 71–78.

Dunham I, Shimizu N, Roe BA, *et al.* 1999. The DNA sequence of human chromosome 22. *Nature* **402**: 489–495.

Jasper H, Benes V, Schwager C, *et al.* 2001. The genomic response of the *Drosophila* embryo to JNK signaling. *Developmental Cell* **1**: 579–586.

Kawarabayasi Y, Hino Y, Horikawa H, *et al.* 2001. Complete genome sequence of an aerobic thermoacidophilic crenarchaeon, *Sulfolobus tokodaii* strain7. *DNA Res* **8**: 123–140.

McClelland M, Sanderson KE, Spieth J, *et al.* 2001. Complete genome sequence of *Salmonella enterica serovar Typhimurium* LT2. *Nature* **413**: 852–856.

Parkhill J, Wren BW, Thomson NR, *et al.* 2001. Genome sequence of *Yersinia pestis*, the causative agent of plague. *Nature* **413**: 523–527.

Parkhill J, Dougan G, James KD, *et al.* 2001. Complete genome sequence of a multiple drug resistant *Salmonella enterica serovar Typhi* CT18. *Nature* **413**: 848–852.

Wright FA, Lemon WJ, Zhao WD, *et al.* 2001. A draft annotation and overview of the human genome. *Genome Biol* **2**: 0025.1–0025.18.

---

Upstream is a compilation of brief reports on papers and press releases of interest to our readers. They represent a personal critical analysis of the original content. If you would like to recommend a paper, or newsworthy item, please contact our Managing Editor.

Copyright © 2001 John Wiley & Sons, Ltd. 
